# Trajectories of insomnia symptoms and insufficient sleep duration in early adolescents: associations with school stress

**DOI:** 10.1093/sleepadvances/zpac018

**Published:** 2022-05-29

**Authors:** Serena V Bauducco, Metin Özdemir, Michael Gradisar, Katja Boersma, Sevgi Bayram-Özdemir

**Affiliations:** Center for Health and Medical Psychology, Örebro University, Sweden; Center for Lifespan Developmental Research, Örebro University, Sweden; Center for Lifespan Developmental Research, Örebro University, Sweden; WINK Sleep, Adelaide, Australia; Sleep Cycle AB, Gothenburg, Sweden; Center for Health and Medical Psychology, Örebro University, Sweden; Center for Lifespan Developmental Research, Örebro University, Sweden

**Keywords:** developmental trends, daily stressors, teenagers, sleep patterns, short sleep, academic stress

## Abstract

**Study Objectives:**

We examined how adolescents’ sleep patterns (i.e. insomnia symptoms and sleep duration) change from early- to mid-adolescence and whether adolescents follow different trajectories. Furthermore, we also examined the characteristics of adolescents within different trajectories, with a specific focus on the role of school-related stress.

**Methods:**

We used three longitudinal waves of questionnaire data collected annually from a sample of Swedish adolescents (*n* = 1294; *M*_age_ = 13.2 [range: 12–15 years], *SD* = .42; 46.8% girls). Using established measures, the students reported on their sleep duration, insomnia symptoms, and perceived school-stress (including stress of school performance, peer and teacher relations, attendance, and school-leisure conflict). We used latent class growth analysis (LCGA) to identify adolescents’ sleep trajectories, and the BCH method to describe the characteristics of the adolescents in each trajectory.

**Results:**

We found four trajectories for adolescents’ insomnia symptoms; (1) low insomnia (69%), (2) low-increasing (17%, ‘emerging risk-group’), (3) high-decreasing (9%), (4) high-increasing (5%; ‘risk-group’). For sleep duration, we found two trajectories; (1) ~8 h sufficient-decreasing (85%), (2) ~7 h insufficient- decreasing (15%; ‘risk-group’). Adolescents in risk-trajectories were more likely to be girls and consistently reported higher levels of school stress, particularly regarding school performance and attending school.

**Conclusions:**

School stress was prominent among adolescents suffering from persistent sleep problems, especially insomnia, and deserves further attention.

**Statement of Significance**

According to adolescents, school stress is one of the primary reasons for poor sleep. Yet, few longitudinal studies have examined whether adolescents who develop sleep problems over time also experience more school-related stress. In this study, we found that, compared to adolescents sleeping well, adolescents at risk for persistent insomnia symptoms and those at risk for insufficient sleep duration from early- to mid-adolescence reported significantly more stress of school performance, school attendance, relationships with peers and teachers, and school-leisure balance. This is the first study to highlight the prominence of specific school-related stressors in adolescents at risk for persistent sleep problems. Future studies should further investigate the direction of effect between school stress and sleep problems in adolescents.

## Introduction

Young people experience increased sleep difficulties and decreased sleep duration as a result of biological and psychosocial changes during adolescence. Adolescents’ sleepiness accumulates more slowly than in children, making them more alert in the evenings, and they develop a preference for later sleep schedules [1]. Spending more time with peers, a higher school-workload, and involvement in extracurricular activities are also squeezed into adolescents’ waking day [2]. Altogether, these changes lead to later bedtimes and, because school schedules impose early wake-up times, shorter sleep duration [1]. However, not all adolescents experience sleep difficulties at the same level. The majority of adolescents experience sleep problems sporadically, yet some suffer throughout their teenage years and even maintain sleep disturbances into adulthood [3, 4]. In turn, sleep problems, even temporary ones, are associated with numerous negative outcomes, including but not limited to, poor school performance, mental ill-health, and worse physical health [5]. Despite an accumulating body of research examining the long-term consequences of sleep problems among adolescents, limited knowledge is available regarding which adolescents develop sleep problems over time. The goal of this study is to identify adolescents at risk for persistent sleep difficulties using a person-oriented approach. Addressing this gap in knowledge is essential to identify potential risk factors and thus develop effective sleep-promoting interventions targeting this age group.

## The Role of School-Stress in Adolescents’ Sleep Difficulties

According to the biopsychosocial and contextual model of sleep [2], biological changes, individual characteristics, and contextual factors contribute to sleep disturbances during adolescence and arguably the transition from childhood to adolescence (e.g. 13–15 years) is particularly demanding. This coincides with one of adolescents’ first big challenges, which is the beginning of upper secondary school. School-related stress, including pressure to perform, attendance, relationships with teachers and peers, and the balance between workload versus free time, is one of the most prevalent daily stressors in early adolescence [6, 7], and one of the primary reasons for poor sleep [8, 9]. Yet, how school stress impacts the development of sleep problems over time has not been widely studied. Cross-sectional studies consistently show that adolescents who report school-related stress also experience worse sleep quantity and quality [10–12]. Interestingly, a recent study focusing on adolescents (age 11–18) in Belgium showed that school pressure negatively impacted adolescents’ sleep quality, but support from teachers and peers was associated with better sleep quality [13]. A few longitudinal studies have examined the role of school-related stress in the development of sleep problems. In particular, academic stress was associated with shorter time in bed over a 6-year period [14], whereas school problems and schoolwork were associated with chronicity of several symptoms of insomnia one year later [15]. Therefore, adolescents might both delay their bedtime because of schoolwork and/or worry about school, thus impacting not only the duration but also the quality of their sleep. In this study, we will expand on previous studies by investigating adolescents’ experience of several school-related stressors in relation to both insomnia symptoms and sleep duration.

## Sleep Difficulties in Adolescence

The most common sleep disturbances in adolescents are symptoms of insomnia and short sleep duration [16]. Between 4% and 39% of adolescents report symptoms of insomnia [17]. Difficulties falling asleep are especially common, since adolescents might be attempting to sleep at a time when they are too alert, to accommodate early school times [18]. Moreover, adolescents might delay their bedtimes because of schoolwork, leisure, or other activities. A consequence of delayed bedtimes and fixed wake-up times on schooldays is insufficient sleep. Insufficient sleep duration in adolescents is defined as less than 7 h sleep/night [19]. Between 14% and 68% of adolescents report insufficient sleep according to a recent study in 24 European and North American countries [20]. Despite this alarmingly high number of adolescents, there is also a significant proportion of adolescents who maintain healthy sleep habits during the teenage years. It is therefore crucial to identify adolescents following normal sleep development versus those at risk for severe and persistent sleep disturbances.

## Sleep Developmental Patterns during Adolescence

Several longitudinal studies showed a normative decrease in sleep duration from childhood to adolescence, and an increase in symptoms of insomnia (e.g. 23–25). A study conducted in a large US-based sample showed that sleep duration decreased steadily from age 13 to 18 years and then increased and stabilized up to the late 20s [21]. However, not all individuals follow this path. One study found large individual variability in adolescents’ sleep decline over time (age 14–17), which translated into 4 different trajectories: “long” sleepers (8.8%), who slept above the recommended 8–10 h and showed a small change over time; “high-normal” sleepers (43.7%), who slept within recommendations at baseline but ended up below by the end of the study; ”low-normal” sleepers (26.7%), who slept below recommendations and reported a steady decline; and “short” sleepers (9.3%), who slept severely below recommendations and declined sharply over time [4]. This highlights that some adolescents may display a “normative decline” whereas others might be a “risk-group” that can be missed when simply looking at mean changes over time. Another study found that for about 1/3 of the adolescents, both a shorter time in bed and difficulties falling asleep that initiated at age 7–9 years persisted into late adolescence (age 16–19) [22]. Moreover, from age ~14, insomnia persisted for 64% of adolescents over 2 years [23], and 42% up to age 21 (3). These studies are useful in showing persistent versus temporary sleep difficulties in adolescents, but they do not inform about *who* the individuals at risk are.

## Previous Study Limitations

A few characteristics of youth displaying normative versus risk-trajectories of sleep problems over time have been identified in several studies, including female sex, lower SES, minority ethnical groups, and later pubertal stage [3, 4, 14, 21, 23–25]. However, these studies, and studies exploring adolescents’ sleep trajectories in general, vary in the quality of the sleep measures used; for example, using “time in bed” versus actual time spent sleeping [14, 22], general “sleep problems” rather than standardized measures of insomnia symptoms [3], and not distinguishing between weekday/weekend sleep [4], although sleep-deprived adolescents typically extended catch up sleep on weekends [18]. Moreover, not all studies have used state-of-the-art statistical techniques to explore whether adolescents follow different developmental trajectories for sleep difficulties (except 4, 14, 28). Thus, they are limited in identifying the factors that distinguish adolescents who are at risk from those following normal development. In particular, studies taking into account individual differences are lacking in young adolescents—an important time to identify a risk-group, when sleep changes occur [1]. Finally, another important limitation is that only a few studies have identified developmentally relevant factors linked to adolescents’ sleep trajectories, such as school-related stress.

Therefore, the specific aims of the present study were to;

1. Explore different sleep trajectories from early to mid-adolescence to potentially identify risk groups showing persistent sleep problems (including insomnia symptoms and insufficient sleep duration) as well as their overlap;
2. Describe school-related stress levels over time as well as demographic characteristics of adolescents in both normal and risk-trajectories.

## Method

### Design and participants

The sample for this study was drawn from a longitudinal study (The Tree Cities’ Study) that aims to understand the development of mental health problems during adolescence using a transdiagnostic perspective. The Tree Cities’ Study was implemented in 18 public schools in three middle-sized cities in Sweden. In each school, 7th grade students (age 13) were targeted in 2014 (Time 1) and were re-assessed at grade 8 (Time 2) in 2015, and grade 9 (Time 3) in 2016. Among the participating 7th grade students (*N* = 1457; 47.3% girls), only those with data on insomnia and sleep duration at Time 1 were included. Thus, the analytical sample of the current study consisted of 1294 students (*M*_age_ = 13.2, *SD* = 0.42; 46.8% girls). The majority of adolescents (78%) had a Swedish background [26] and the socioeconomic status (family affluence as perceived by the child) for the sample was slightly higher than a large representative sample of Swedish adolescents (*M*_*SES*_ = 6.72, *SD* = 1.60 vs. *M*_*SES*_ = 6.28, *SD* = 1.67) and indicate high affluence (scores 6–9) [27]. Most adolescents (72.8%) reported living with both parents, and 14% reported switching between mother and father.

## Attrition Analysis

Retention rate from Time 1 to Time 3 was 82.8% in the analytic sample (*N* = 1071). Of the adolescents participating at Time 1, 17.2% (*N* = 226) had dropped out of the study at Time 3. We ran a logistic regression analysis to examine if dropout was systematic, predicting attrition at Time 3 (1 = attrition; 0 = retention). Predictors included demographic variables (i.e. sex, immigrant background, and SES), school-related stress (i.e. peers and teacher relations, school performance, attendance, school/leisure conflict), and sleep variables (i.e. sleep duration and insomnia) at Time 1. The results showed that immigrant background (Wald = 8.36, *p* = .004, OR = 1.73, CI: 1.19–2.51), and insomnia symptoms (Wald = 4.44, *p* = .03, OR = 1.04, CI: 1.00–1.08) were significant predictors of attrition at Time 3 (Nagelkerke *R*^2^ = .059). That is, adolescents with an immigrant background and higher levels of insomnia had greater likelihood of dropping out from the study at Time 3.

## Procedure

The data were collected annually during the spring term. Adolescents completed the questionnaire (paper and pen) in the classroom during school hours. Trained test leaders administered the surveys allowing students 90 min to complete the questionnaires. Each class received 300 Swedish crowns in recognition of participation. Before participation, active consent from students and passive consent from parents were received. Passive consent has shown to increase participation rate and to limit sampling bias [28, 29]. Moreover, students filled out a consent form in the classroom on the day of data collection and were informed about confidentiality, that participation was voluntary and that they could choose to withdraw from the study at any time. Adolescents who did not speak Swedish or reported other difficulties with understanding written language at the time of data collection were excluded from the study. The project was approved by the Regional Ethical Board in Uppsala, Sweden.

## Measures

### Sleep duration

Weekday sleep duration was estimated from adolescents’ self-reported bedtime (“What time do you usually go to bed on school days?”), wake-time (“What time do you usually wake up on school days?”), and sleep onset latency (“On school nights, after you go to bed, about how long does it take for you to fall asleep?”) during the past 2 weeks. These items were drawn from the School Sleep Habits Survey [30], which has shown good validity when compared to objective sleep measures [31, 32]. Weekday sleep duration was calculated as the interval between bed- and wake-time, minus sleep onset latency.

### Insomnia

We used the 7-item Insomnia Severity Index (ISI) to measure symptoms of insomnia among adolescents [33]. Items cover difficulties falling asleep, staying asleep, and waking up too early as well as perceived satisfaction with sleep, interference with daytime functioning and worry about sleep. The time frame in this study was changed from 2 weeks (in the original) to the last 6 months to match how other complaints were measured in the rest of the survey. Response option ranged from 0 to 4, with higher scores indicating more severe problems and a possible total score of 28. The recommended cutoff for clinical insomnia in adolescents is a total score of 9 [34]. The scale has been shown to be reliable and valid in adolescent populations [34]. In the present study, Cronbach’s alpha was .82 at T1, .84 at T2, and .87 at T3.

### School-related stress

The short version of the Adolescent Stress Questionnaire, ASQ [7, 35] was used to measure adolescents’ experiences of school stress. The ASQ is a stressor checklist measuring adolescents’ daily stressors (e.g. school, family, romantic relationships). In this study, we only focus on school-related stress and included the following subscales; stress in peer relationship (four items; “*pressure to fit in with peers*”), conflicts with teachers (three items, “*getting along with your teachers*”) (Cronbach’s alpha at T1.81, T2.85, T3.89), school performance (three items, “*keeping up with school work*”) (Cronbach’s alpha at T1.83, T2.83, T3.88), school-leisure conflict (three items, “*not getting enough time for leisure*”) (Cronbach’s alpha at T1.83, T2.86, T3.87), and attendance (two items, “*getting up early in the morning to go to school*”) (Cronbach’s alpha at T1.78, T2.78, T3.79) in the last 6 months. Adolescents rated how stressful each event was on a 5-point Likert scale, ranging from 1 (*not at all/it has not happened*) to 5 (*very stressful*). These subscales have been validated and showed good psychometric properties [35].

### Demographics

Adolescents were asked about their sex (male/female) and age. Socioeconomic status (SES) was assessed through four questions about family affluence from the Family Affluence Scale (FAS-II), which was used in the Health Behavior in School-aged Children (HBSC) [36]. Questions about adolescents’ immigrant background included place of birth and parents’ place of birth (including Sweden, Scandinavia, Europe and outside Europe). Immigrant background was defined as being born outside of Sweden or being born in Sweden with both non-Swedish parents according to Statistics Sweden [26].

## Data Analysis

We followed a two-step procedure to identify different sleep-pattern trajectories [37]. In Step 1, we estimated a latent curve growth model to examine how, on average, adolescents’ sleep problems changed over time, and to examine whether there was a significant variation in intercept (initial level) and slope (change over time) of sleep problems. In Step 2, we used latent class growth analysis (LCGA) to identify adolescents with different sleep trajectories. To determine the number of trajectories that best fit the data we used the Bayesian Information Criterion (BIC [38],), entropy (i.e. classification accuracy [39],), Lo–Mendell–Rubin test (LMR [40],), and the proportion of adolescents in each group trajectory [37]. Specifically, we chose the cluster solution that had the lowest BIC, highest entropy, and nonsignificant LMR (i.e. model fit improvement from *n* − 1 to *n* classes) [37]. We analyzed trajectories for insomnia symptoms and sleep duration separately.

After establishing the different sleep trajectories, we compared adolescents in each trajectory on sex distribution, immigrant background, SES, and school-related stress (i.e. stress of school performance, teacher conflict, attendance, school vs. leisure conflict, and peer relationships). School stress was examined at each time point because it can fluctuate over time [41]. The BCH method in Mplus was used to compare the trajectories. This method did not affect the trajectories established in the LCGA model [42]. The BCH method estimates the mean of the variables of interest (e.g. stress of school performance) across trajectories. Chi-square comparisons are performed to assess overall difference among trajectories and pairwise comparisons among trajectories (if >2) [42]. As these analyses do not provide effect sizes of the overall difference, we calculated these by hand.

Data were analyzed in Mplus [43]. We handled missing data in Mplus using full information maximum likelihood (FIML). Insomnia model: 4 missing data patterns, covariance coverage ranged between 0.76 and 1.00. Sleep duration model: 4 missing data patterns, covariance coverage ranged between 0.75 and 1.00. The covariance coverage was well above the recommended 0.10 to reliably use FIML. Moreover, FIML is a superior method compared to mean imputation, listwise deletion or pairwise deletion, as it provides more reliable standard errors [44, 45].

## Results

### Descriptive statistics

Means and standard deviations of the sleep variables (i.e. insomnia and sleep duration) and school-related stress (i.e. peers and teacher relations, school performance, attendance, school/leisure conflict) are presented in Table 1.

**Table 1.** Means and standard deviations among the study variables

Sleep variables	*Mean* (*SD*)
Sleep duration T1	8:07 h (1:07)
Sleep duration T2	7:57 h (1:04)
Sleep duration T3	7:43 h (1:07)
Insomnia T1	5.05 (4.74)
Insomnia T2	5.29 (4.85)
Insomnia T3	6.25 (5.61)
School stressors	
School performance T1	2.44 (1.07)
School performance T2	2.88 (1.24)
School performance T3	2.81 (1.50)
Teacher conflict T1	1.49 (0.48)
Teacher conflict T2	1.58 (0.65)
Teacher conflict T3	1.56 (0.71)
Attendance T1	1.87(1.01)
Attendance T2	2.02 (1.18)
Attendance T3	2.08 (1.31)
School/leisure conflict T1	2.07 (1.02)
School/leisure conflict T2	2.36 (1.29)
School/leisure conflict T3	2.26 (1.37)
Peer stress T1	1.36 (0.34)
Peer stress T2	1.33 (0.30)
Peer stress T3	1.34 (0.35)

*Note.* Insomnia scores range (0–28), School-related stress scores range (1–5).

## Sleep Changes Over Time

Two separate latent growth curve models were estimated to examine whether and how adolescents’ insomnia symptoms and sleep duration changed during adolescence (i.e. from 13 to 15 years old; Table 2). The linear latent growth curve model for insomnia yielded a good model fit χ ^2^ [1] = 10.96, *p* < .01, CFI = .98, RMSEA = 0.09, 95% CI for RMSEA 0.05–0.14, SRMR = 0.02. The mean of the slope was positive and statistically significant, which indicates that adolescents reported increasing symptoms of insomnia from early to mid-adolescence. The variances of the slope and the intercept were both statistically significant, which indicates that adolescents differed both in the level of insomnia symptoms at T1 and in how they changed from T1 to T3. These results suggest that there might be unique subgroups of adolescents who follow different trajectories in insomnia symptoms over time.

The linear latent growth curve model for sleep duration also yielded a good model fit χ ^2^ [1] = 0.05, *p* = .48, CFI = 1, RMSEA = 0.0, 95% CI for RMSEA 0.00–0.06, SRMR = 0.006. The mean of the slope was negative and statistically significant, which indicates that adolescents’ sleep duration, on average, significantly decreased over time. The variances of the slope and the intercept were both significant, indicating that adolescents differed both in the level of sleep duration at T1 and in how they changed from T1 to T3. These results suggest that there might be unique subgroups of adolescents who follow different growth trajectories with regard to their sleep duration.

## Insomnia Subgroup Trajectories

We used latent class growth models (LCGMs) to identify subgroups of adolescents who follow different trajectories of insomnia over time. As shown in Table 3, BIC and entropy improved from one-class to a five-class solution. Besides, the LMR-LRT test was not significant for the 5-class solution, indicating that there was no significant improvement in model fit to the data from a 4-class to a 5-class solution. Therefore, the 4-class solution was retained.

**Table 2.** Model fit, intercept, and growth estimates for the linear change model in insomnia and sleep duration (in hours) over two years

	Model fit statistics							Intercept		Slope	
	χ ^2^(df)	*p*	CFI	RMSEA	90% CI	*p*	SRMR	*Mean*	Variance	*Mean*	Variance
Insomnia	10.96 (1)	<.001	0.98	0.09	.046,.138	.065	0.019	4.94***	12.88***	0.68***	2.21**
Sleep duriation	0.05 (1)	.48	1	0.00	.00,.065	.869	0.006	8.13***	.73***	–.22***	.10**

*Note. N* = 1294, Estimator = MLR, ***p <.0.001

**Table 3.** Model fit statistics for analysis of growth trajectories of insomnia and sleep duration (in hours) over two years

	Entropy	AIC	BIC	Adj. BIC	Adj. LRT	*p*
Insomnia						
2 cluster	0.877	20162.42	20203.74	20178.33	1070.44	<.001
3 cluster	0.827	19928.44	19985.26	19950.32	229.31	.024
**4 cluster**	0.846	19740.86	19813.18	19768.71	184.98	.005
5 cluster	0.798	19668.35	19756.16	19702.16	75.02	.655
6 cluster	0.812	19591.52	19694.83	19631.3	79.15	.250
Sleep duration						
**2 cluster**	0.831	9974.83	10016.15	9990.74	602.38	<.001
3 cluster	0.784	9811.84	9868.66	9833.72	161.475	0.517
4 cluster	0.792	9667.33	9739.65	9695.18	143.82	0.012
5 cluster	0.803	9612.35	9700.16	9646.16	58.27	0.297
6 cluster	0.793	9561.99	9665.3	9601.77	50.29	0.536

**Figure 1.**
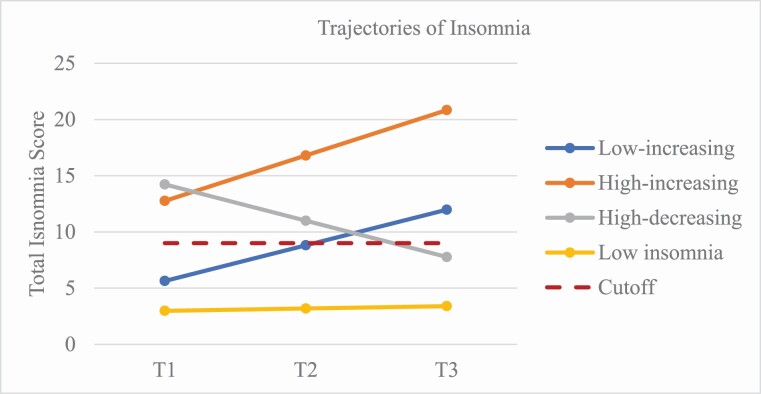
Trajectories of insomnia over time. *Note. Cutoff = a total score 9 on the Insomnia Severity Index (ISI)* [35]

The first class (*low-increasing*) included 17.2% of the sample (see Table 4). Adolescents in this class reported low levels of insomnia symptoms at T1 and subthreshold symptoms at T3. The second class (*high-increasing*) included 4.6% of the sample and represented the risk group. Adolescents in this trajectory class reported subthreshold symptoms of insomnia at T1 and increased to the upper limit of moderate insomnia at T3. The third class (*high-decreasing*) represented 9% of the sample. Adolescents reported subthreshold symptoms of insomnia at T1 and their insomnia symptoms decreased substantially by T3. The final trajectory class (*low insomnia*) represented 69.2% of the sample. Adolescents in this class reported low levels of insomnia symptoms at T1 and showed a slight increase in their symptoms over time. The four trajectories and the estimated mean changes are shown in Figure 1.

**Table 4.** Growth estimates and cluster sizes for insomnia and sleep duration

Cluster name	Latent classes		Intercept		Slope	
	*n*	Proportions, %	*Mean*	*p*	*Mean*	*p*
Insomnia						
Low-increasing	222	17.2	5.64	<.001	3.17	<.001
High-increasing	60	4.6	12.76	<.001	4.04	<.001
High-decreasing	116	9.0	14.23	<.001	–3.24	<.001
Low insomnia	896	69.2	2.98	<.001	0.21	.012
Sleep duration						
Short-decreasing	196	15.1	6.72	<.001	–.33	<.001
Adequate-decreasing	1098	84.9	8.41	<.001	–.20	<.001

## Sleep Duration Trajectories

LCGMs analysis for sleep duration yielded two different trajectories (Table 3). The class solutions fit indices BIC and entropy improved from one-class to three-class. However, the LMR-LRT test was not significant for the 3-class solution, suggesting that the 2-class solution fits the data better. The LMR-LRT test was significant for the 4-class solution, but entropy was worse than the 2-class solution. Moreover, the 4-class solution yielded two very small classes, 1% and 4% of the sample, which were not theoretically justifiable. For the sake of parsimony we retained the 2-class solution.

**Figure 2.**
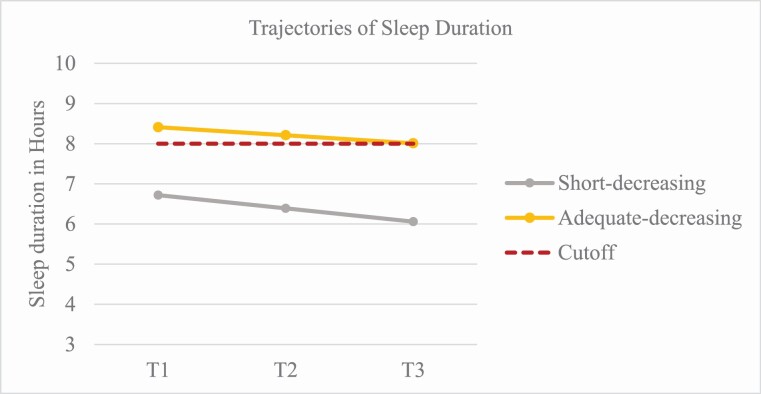
Trajectories of sleep duration over time. *Note.* Cutoff = 8 hours/night [20]

The first class (*sufficient-decreasing*) included 84.9% of the sample (see Table 4). Adolescents in this trajectory showed a statistically significant decline in sleep duration from an average of 8.4–8.0 h of sleep/weeknight and, therefore, still within the recommended range for this age group [19]. The second trajectory (*insufficient-decreasing*) included 15.1% of the sample and represented the risk group. Adolescents in this trajectory reported a weekday sleep duration of 6.7 h/night at T1 (<7 h; insufficient sleep duration) to 6.1 h/night at T3 (insufficient). The two trajectories and the estimated mean changes are shown in Figure 2.

## Overlap Between Insomnia and Insufficient Sleep Trajectories

The insomnia risk-group (i.e., *high-increasing*) included 45% of the sleep duration risk-trajectory and 55% of the normative sleep trajectory class. The sleep duration risk-group included 13.8% of adolescents in the insomnia high-increasing trajectory class, 20.9% in the decreasing trajectory, 26% in the low-increasing and the largest proportion, 39.3%, was from the stable-low insomnia trajectory class.

## Adolescents’ Sleep Trajectories: Differences and Similarities in Demographics and School-Related Stress

We examined whether adolescents in each sleep trajectory of insomnia symptoms and sleep duration (see Table 4) differed from each other in demographic characteristics (i.e. sex, immigrant background, and SES), and daily stressors in the school context (i.e. peers and teacher relations, school performance, attendance, and school/leisure conflict).

**Differences and similarities among adolescents in different insomnia trajectories.** In general, girls were underrepresented in the *low insomnia* trajectory (40.3%) whereas they were the majority in the *high-increasing* (77%), *high-decreasing* (56.5%), and *low-increasing* (57.3%) insomnia trajectories (see Table 5). There were no differences in immigrant background among adolescents in different trajectories, but adolescents in the *low-increasing* trajectory reported lower SES as compared to the *low-insomnia* trajectory.

**Table 5.** Differences and Similarities in Demographics and School-Related Stressors at T1, T2, and T3 by Insomnia Trajectories

	Low insomnia *M* (*SD*)/%	High increasing *M* (*SD*)/%	High decreasing *M* (*SD*)/%	Low increasing *M* (*SD*)/%	χ ^2^ (df)	*p*	ω ^2^
Sex (girl)	40.3%^a^	77%^b^	56.5%^c^	57.3%^c^	49.45 (3, 1294)	<.001	.03
Immigrant background	22.8%	23.6%	25.8%	17%	3.14 (3, 1294)	.370	.00
SES	6.82^a^(.06)	6.43^ab^(.24)	6.57^ab^(.20)	6.48^b^(.13)	8.39 (3, 1294)	<.050	.00
School performance T1	2.17^a^(.03)	3.37^b^(.15)	3.40^b^(.13)	2.67^c^(.08)	178.48 (3, 1294)	<.001	.12
School performance T2	2.58^a^(.04)	4.03^b^(.15)	3.53^c^(.13)	3.31^c^(.08)	174.10 (3, 1294)	<.001	.12
School performance T3	2.49^a^(.04)	3.93^b^(.18)	3.36^c^(.19)	3.45^c^(.10)	139.29 (3, 1294)	<.001	.10
Teacher conflict T1	1.34^a^(.02)	2.22^b^(.13)	1.99^b^(.10)	1.58^c^(.06)	105.48 (3, 1294)	<.001	.07
Teacher conflict T2	1.41^a^(.02)	2.44^b^(.17)	1.97^c^(.10)	1.80^c^(.07)	90.67 (3, 1294)	<.001	.06
Teacher conflict T3	1.34^a^(.03)	2.34^b^(.20)	1.85^b^(.15)	2.01^b^(.08)	94.47 (3, 1294)	<.001	.07
Attendance T1	1.60^a^(.03)	2.84^b^(.18)	2.83^b^(.14)	2.13^c^(.09)	165.75 (3, 1294)	<.001	.11
Attendance T2	1.73^a^(.03)	3.51^b^(.19)	2.42^c^(.14)	2.47^c^(.09)	160.12 (3, 1294)	<.001	.11
Attendance T3	1.74^a^(.04)	3.70^b^(.20)	2.49^c^(.18)	2.70^c^(.10)	181.47 (3, 1294)	<.001	.12
School/leisure conflict T1	1.85^a^(.03)	3.15^b^(.17)	2.75^b^(.12)	2.22^c^(.08)	129.38 (3, 1294)	<.001	.09
School//leisure conflict T2	2.11^a^(.04)	3.46^b^(.19)	2.77^c^(.15)	2.73^c^(.09)	103.16 (3, 1294)	<.001	.07
School/leisure conflict T3	1.98^a^(.04)	3.49^b^(.20)	2.44^c^(.18)	2.85^c^(.10)	112.61 (3, 1294)	<.001	.08
Peers T1	1.25^a^(.02)	2.11^b^(.14)	1.65^c^(.08)	1.42^d^(.05)	77.92 (3, 1294)	<.001	.05
Peers T2	1.22^a^(.02)	1.81^b^(.12)	1.61^bc^(.09)	1.49^c^(.05)	68.77 (3, 1294)	<.001	.05
Peers T3	1.21^a^(.02)	1.94^b^(.18)	1.51^b^(.11)	1.60^b^(.06)	67.57 (3, 1294)	<.001	.05

*Note.* SES = socioeconomic status.

Different letters in the superscript indicate a significant difference between trajectories. For example, stress of school performance at T1 was significantly lower in the “low insomnia” (a) trajectory as compared to all other trajectories, but did not differ between the “high-increasing” (b) and “high-decreasing” (b).

Adolescents in the *low-insomnia* trajectory clearly showed lower levels of school-stress (i.e. peers and teacher relations, school performance, attendance, and school/leisure conflict) as compared to the other trajectories. In contrast, adolescents in the *high-increasing* trajectory reported consistently higher levels of school-related stress compared to adolescents in the *low-insomnia* trajectory.

Finally, at T1, the adolescents in the *high-decreasing* insomnia symptoms trajectory did not differ from their peers in the *high-increasing* trajectory regarding school stress. However, adolescents in these opposing trajectories reported significantly different levels of school-related stress at T3 (except for teacher and peer relations). That is, the high-decreasing group trajectory reported less stress of school attendance, performance and school/leisure conflict compared to the high-increasing group trajectory by the end of the study. Moreover, compared to the *low-increasing* trajectory, the adolescents in the *high-decreasing* trajectory showed significantly higher levels of school-related stress at T1 but no significant differences at T3. Therefore, daily school-stress may help to discern some of the risk trajectories from each other but not all.

**Differences and similarities among adolescents in different sleep duration trajectories.** Girls represented the majority (67.7%) in the *insufficient-decreasing* sleep duration trajectory. There were no differences in immigrant background and SES. At all three time points, adolescents in this risk-trajectory reported higher levels of school stress (except peer-related stress) compared to those in the *sufficient-decreasing* sleep duration trajectory (see Table 6). Therefore, adolescents at risk for developing sleep deficit over time present with higher stressor-load as compared to their peers obtaining sufficient sleep.

**Table 6.** Differences and Similarities in Demographics and School-Related Stressors at T1,T2,T3 by Sleep Duration Trajectories

	Short *M* (*SD*)	Adequate *M* (*SD*)	χ ^2^(df)	*p*	ω^2^
Sex (Girl)	67.7%	42.9%	30.23(1,1294)	<.001	.06
Immigrant background	20.1%	22.4%	0.37(1,1294)	.544	.00
SES	6.57(.14)	6.74(.05)	1.15(1,1294)	.283	.00
School performance T1	2.87(.10)	2.36(.03)	22.84(1,1294)	<.001	.02
School performance T2	3.42(.11)	2.78(.04)	29.15(1,1294)	<.001	.02
School performance T3	3.23(.13)	2.74(.04)	12.11(1,1294)	.001	.01
Teacher conflict T1	1.79(.07)	1.43(.02)	21.75(1,1294)	<.001	.02
Teacher conflict T2	1.91(.09)	1.52(.02)	16.20(1,1294)	<.001	.01
Teacher conflict T3	1.83(.09)	1.51(.03)	9.37(1,1294)	.002	.01
Attendance T1	2.41(.10)	1.77(.03)	34.24(1,1294)	<.001	.03
Attendance T2	2.75(.12)	1.90(.03)	45.29(1,1294)	<.001	.03
Attendance T3	2.70(.13)	1.97(.04)	27.92(1,1294)	<.001	.02
School/leisure conflict T1	2.47(.10)	1.99(.03)	18.89(1, 1294)	<.001	.01
School//leisure conflict T2	2.80(.11)	2.28(.04)	18.63(1, 1294)	<.001	.01
School/leisure conflict T3	2.66(.13)	2.19(.04)	11.47(1, 1294)	.001	.01
Peers T1	1.52(.06)	1.33(.02)	8.38(1, 1294)	<.01	.02
Peers T2	1.47(.06)	1.31(.02)	5.60(1, 1294)	<.05	.01
Peers T3	1.39(.06)	1.34(.02)	0.53(1, 1294)	.465	.00

*Note.* SES = socioeconomic status.

## Discussion

Adolescents’ sleep undergoes normative changes. Yet, some adolescents experience sleep problems sporadically, whereas others develop persistent problems. However, there is a lack of knowledge on *how* sleep problems develop and *who* these adolescents are. In this study, we examined how adolescents’ sleep patterns (i.e. insomnia symptoms and sleep duration) change from early- to mid-adolescence and whether adolescents follow different trajectories. Furthermore, we also examined the characteristics of adolescents with different trajectories, with a specific focus on school-related stress.

## Trajectories of Insomnia Symptoms and Sleep Duration

On an average, adolescents reported increasing symptoms of insomnia over time, yet we found large variation suggesting that not all adolescents followed the same developmental path. We found four insomnia trajectories; two “low-risk” trajectories, one “emerging risk-group”, and one “risk” trajectory. Most adolescents followed the *low insomnia* trajectory (69.2%) over 2 years, whereas 9% were classified as *high-decreasing,* moving from subclinical insomnia to good sleep by the end of the study. The emerging risk-group was the *low-increasing* trajectory (17.2%), with adolescents reporting symptoms below the cutoff at age 13 and above by age 15. The risk group was the *high-increasing* insomnia symptoms trajectory (4.6%)—with adolescents reporting symptoms above the cutoff and yet still increasing throughout the study. Previous studies have shown a high persistence of clinical symptoms [23, 46], and a general increase up to age 25 [46]. However, these studies have not focused on individual differences in the development of insomnia over time. Using a person-oriented approach, the present study uncovered one group of adolescents who remit from their insomnia, which provides an opportunity to understand potential protective factors.

Similarly, not all adolescents’ sleep duration changed at the same rate. We found two distinct trajectories—one low-risk and one high-risk group. The majority of adolescents (85%) obtained sufficient sleep throughout the study (≥8 h), whereas 15% reported insufficient sleep (<7 h) and a sharp decrease over time. This is in contrast with a similar study [4], where Canadian adolescents followed four different trajectories. All four declined over three years, and only one (8%) stayed above the recommended sleep duration of 8–10 hr/night. This difference might be due to the slightly older age group (14–16 yrs) and to the fact that European adolescents have been found to sleep more than their peers in USA and Canada [18]. Similar to insomnia, studies with older adolescents show that insufficient sleep worsens over time [25, 46]. In turn, these sleep disturbances have negative implications for adolescents’ daytime functioning [5]. Given that adolescents follow different trajectories in their sleep duration and quality, it is essential to examine the potential reasons behind these variations.

## Differences in School Stress among Sleep Trajectories

In our study, we aimed to describe the experience of school-related stress among normal and problematic sleep trajectories, which is an understudied factor in adolescent sleep research [2]. As expected, school stress was higher among adolescents with persistent symptoms of insomnia and those with insufficient sleep duration. This finding adds to previous cross-sectional and longitudinal studies [10–15] and shows that adolescents with persistent sleep problems might experience concurrent high levels of school-related stress, making school stress an important aspect to consider in the promotion of adolescents’ sleep health. The difference in school stress among trajectories of insomnia symptoms were medium-to-large. Although it is not always possible to remove the source of stress, such as a school transition, it may be possible to help adolescents better cope with the stress [47]. For example, interventions targeting school stress have been tested and successfully improved adolescents’ repertoire of coping strategies, yet no changes in sleep were reported [48]. However, the lack of impact on sleep might be due to the fact that the intervention was universal and not all adolescents were stressed or reported problems sleeping to begin with. On the other hand, there is evidence that better sleep promotes better coping to handle stressors [49]. Although longitudinal, our study cannot ascertain directionality in the association between sleep and school stress, and it is possible that a bidirectional association is in play. That is, adolescents who sleep poorly might also be more vulnerable to stress, as compared to peers who sleep well [49]. Therefore, future studies, including intervention studies, should investigate the direction of effect between stress and sleep disturbances to establish whether changes in perceived stress may lead to changes in sleep and/or vice versa.

Despite a clear pattern of distinction between adolescents at high versus low risk for sleep problems in school stress levels, the current study showed no clear distinction between the *low-increasing* and *high-decreasing* insomnia symptom trajectories. That is, adolescents starting with low but increasing insomnia symptoms over time reported significantly less school-related stress at age 13 compared to those with decreasing symptoms, but these two groups did not significantly differ from each other at age 15. Thus, suggesting that school-related stress might not contribute to the rise or decline in insomnia symptoms in the divergent trajectories. These findings seem contradictory to the theoretical view of life stressors as precipitating factors for the onset of insomnia [50] but might be due to a missing link: how adolescents deal with stress and downregulate arousal, as suggested by the Psycho-bio-behavioral model of vulnerability to insomnia [51]. In line with this model, a recent study found that heightened emotional reactivity and reduced emotion regulation capacity strongly predicted the development of clinical insomnia from childhood to adolescence [52]. Future studies are needed to explore the mechanisms explaining the link between school-stress and sleep disturbances. Nevertheless, adolescents reporting sufficient sleep duration and low insomnia symptoms over time consistently reported lower levels of school-related stress. This suggests that school stress might be an important target for promoting sleep health in adolescents.

## Implications for Interventions and Potential Targets

Preventive interventions can be targeted to a specific risk-group or include all adolescents, for instance via school-based programs [53]. In the present study, adolescents at risk for insomnia symptoms did not necessarily report insufficient sleep, and vice versa. In fact, the majority (55%) in the *high-increasing* insomnia symptoms trajectory were adolescents reporting adequate sleep duration. Similarly, the largest proportion of adolescents reporting insufficient sleep over time came from the *low-insomnia* group (39.3%). Moreover, although we did not directly test for differences in characteristics across sleep problems, adolescents in the *high-increasing* insomnia symptoms group appear to report a particularly high stressor load. Therefore, it might be easier to identify adolescents at risk for persistent insomnia symptoms as compared to insufficient sleep, for targeted interventions [54]. On the other hand, a broader approach of universal interventions seems justified for insufficient sleep duration, as even adolescents in the largest “low-risk” group showed a declining trend over time. As to what to target, similar aspects of school-related stress emerged as central for both adolescents at risk for insomnia and insufficient sleep.

A novel aspect of the present study was the exploration of various school-related stressors. First, school performance was a common stressor among risk-trajectories, in line with qualitative evidence of adolescents’ perceived reasons for poor sleep [8, 9]. Second, school attendance was also prominent among risk trajectories and may be largely related to difficulties waking up in the morning [7], because of adolescents’ delayed sleep timing [1]. Third, a perceived lack of free time was also more prevalent among the *high-increasing* insomnia group. This in turn could be linked to “bedtime procrastination”, defined as delaying bedtime without external reasons to fulfill the need for free time activities [55]. This need has been described by adolescents as a reason for not prioritizing sleep [9]. Contrary to expectations, however, in this study the difference in school-leisure conflict between the two sleep duration trajectories was small. Yet, this was true for all school-related stressors, where effect sizes were larger for comparisons among insomnia symptoms as compared to sleep duration trajectories. A reason for this might be that much of the decline in sleep duration is due to the biological changes occurring in adolescents, whereas contextual factors such as school stress explain a smaller portion [56]. Finally, in the present study, relational stressors (i.e. peers and teachers) did not consistently distinguish adolescents in the high-risk trajectories. Yet, recent cross-sectional findings showed that support from peers and teachers was protective for adolescents’ sleep [13], and this seems true for the adolescents in the *low-insomnia* trajectory in the present study. To conclude, in addition to interventions targeting individual skills (e.g. stress management), targeting the school context (e.g. start times, workload) might be needed to promote sleep health in adolescents.

In addition to school-related stress, adolescents in high- versus low-risk trajectories differed from each other with regard to sex. Girls were overrepresented in the risk-trajectories of both insomnia symptoms and insufficient sleep. This is in line with previous studies, where girls have consistently been found at higher risk for insomnia, starting from puberty [57]. However, sex differences in insufficient sleep have been reported inconsistently across countries and some studies have found boys at higher risk for short sleep [18]. These differences should be investigated further, also considering that girls report more school stress than boys [6, 58], and this was significantly higher among risk-trajectories compared to good sleepers.

## Limitations and Strengths

This study has a number of strengths and limitations. Although we did not include objective measures of sleep (e.g. actigraphy), self-reported sleep has been found to be valid compared to actigraphy [32], and is cost-effective in large, longitudinal studies. Moreover, guidelines are mainly based on self-reports and there is value in how adolescents perceive their sleep [59]. Another limitation is that we did not measure pubertal status, which might underlie differences in sex distribution among the trajectories (e.g. [60]). This study has also a number of strengths, including the large sample size, the longitudinal design, and examining heterogeneity in the overtime change patterns, which allowed to identify risk-trajectories as well as the characteristics associated with the adolescents at risk. This is particularly relevant for planning interventions as it gives information about *who* to target (e.g. universal vs. indicated prevention); high stressor load. Moreover, studying these trajectories from early adolescence is important to inform preventive efforts, as this is a sensitive time for the development of sleep problems as well as psychopathology [57]. Finally, school stress has received little attention in previous longitudinal studies but was clearly prominent in adolescents with persistent insomnia symptoms and insufficient sleep compared to good sleepers. Future studies should further explore the role of school-related stress in the development of poor sleep, including the role of adolescents’ coping in the face of school-stress, and specific aspects of the school environment (e.g. class climate and bullying).

## Conclusions

This study shows a complex picture in the development of insomnia from early- to mid-adolescence, with four distinct trajectories, and thus provides important information on *when* to intervene to prevent insomnia symptoms. For the *low-increasing* group, the beginning of adolescence might be a window of opportunity, but for the *high-increasing* trajectory it might be too late. Piecing together studies from childhood shows that a high-stable insomnia risk-group can be identified earlier [22, 52, 61]. The trajectories of sleep duration, on the other hand, confirm its status as a public health concern and the need for universal interventions at the beginning of adolescence. Using a person-oriented perspective, this study was able to show similar profiles for adolescents at risk for persistent insomnia symptoms and insufficient sleep duration. In particular, school stress was highly prominent in adolescents at risk for poor sleep quality and quantity and deserves further attention.

## Data Availability

The data underlying this article cannot be shared publicly due to the agreement made with the individuals that participated in the study. The data will be shared on reasonable request to the corresponding author.

## Dislosure Statement

Michael Gradisar is CEO at WINK Sleep Pty Ltd.
